# Osteochondral Regeneration with a Scaffold-Free Three-Dimensional Construct of Adipose Tissue-Derived Mesenchymal Stromal Cells in Pigs

**DOI:** 10.1007/s13770-017-0091-9

**Published:** 2017-11-15

**Authors:** Daiki Murata, Shizuka Akieda, Kazuhiro Misumi, Koichi Nakayama

**Affiliations:** 1 Department of Veterinary Clinical Science, Joint Faculty of Veterinary Medicine, Kagoshima University, 21-24 Korimoto 1-chome, Kagoshima, 890-0065 Japan; 2 Cyfuse Biomedical K.K, 1-1 Maidashi 3-chome, Higashi-ku, Fukuoka, 812-8582 Japan; 3 Department of Regenerative Medicine and Biomedical Engineering, Faculty of Medicine, Saga University, Honjyo 1-chome, Honjyo-cho, Saga, 840-8502 Japan

**Keywords:** Osteochondral regeneration, Scaffold-free, Three-dimensional construct, Mesenchymal stromal cells, Adipose tissue

## Abstract

Osteochondral lesion is a major joint disease in humans. Therefore, this study was designed to investigate the regeneration of articular cartilage and subchondral bone, using three-dimensional constructs of autologous adipose tissue-derived mesenchymal stromal cells without any biocompatible scaffolds. Mesenchymal stromal cells were harvested by liposuction from seven pigs, isolated enzymatically, and expanded until construct creation. The pig models had two osteochondral defects (cylindrical defects with a diameter of 5.2 mm and a depth of 5 mm) in one of their patello-femoral grooves. A columnar structure consisting of approximately 770 spheroids of 5 × 10^4^ autologous mesenchymal stromal cells were implanted into one of the defects (implanted defect), while the other defect was not implanted (control). The defects were evaluated pathologically at 6 months (in three pigs) and 12 months (in five pigs) after implantation. At 6 months after surgery, histopathology revealed active endochondral ossification underneath the plump fibrocartilage in the implanted defects, but a deficiency of fibrocartilaginous coverage in the controls. At 12 months after surgery, the fibrocartilage was transforming into hyaline cartilage as thick as the surrounding normal cartilage and the subchondral bone was thickening in the implanted defects. The histological averages of the implanted sites were significantly higher than those in the control sites at both 6 and 12 months after surgery. The implantation of a scaffold-free three-dimensional construct of autologous mesenchymal stromal cells into an osteochondral defect can induce regeneration of hyaline cartilage and subchondral bone structures over a period of 12 months.

## Introduction

Osteochondral lesion, defined as cartilage degradation and subchondral bone sclerosis/deformity, is a major joint disease that typically develops into Osteoarthritis, and the associated disability can decrease quality of life in humans [1]. The osteochondral autografts (mosaicplasty) from the non-load-bearing sites to the deteriorated sites (the loading-bearing sites) showed favorable outcomes (clinical improvement in 79–94% of patients with osteoarthritis) [2]. However, the loss of cartilage and bone in the non-load-bearing sites was inevitable [3]. The implantation of artificial bone and autologous chondrocytes seeded into a collagen scaffold has also shown favorable restoration of bone and cartilage [4, 5]. It is well known that bone is self-restorative, because its vascularity and cellularity are convenient to degrade the artificial bone (osteoclastic system) and later to reform a new bone matrix (osteoblastic system) [6, 7]. In contrast, the articular cartilage has been suggested to be less restorative, because it is only formed by chondrocytes, which both degrade and regenerate the special matrix [6, 7]. Further, the collagen scaffold remaining in the implanted sites for long periods can prevent the regeneration of hyaline cartilage, but can also promote its replacement to fibrous cartilage [8]. Based on these theories, it is possible that artificial scaffold can be unfavorable in order to regenerate articular cartilage; therefore, investigation of cartilage regeneration by using scaffold-free construct of cells is necessary. In fact, there is still no ideal biomaterial for scaffold construction, although many studies have reported the successful use of various biomaterials. Furthermore, many concerns remain that need to be solved, including immunogenicity [9, 10], long-term safety of scaffold degradation products [11], and risk of infection or transmission of disease.

Previous studies on autologous chondrocyte implantation have presented that few chondrocytes can be isolated from a small piece of normal cartilage [4], and that some of them can be dedifferentiated during the culture passage [12]. To resolve these problems, cartilage regeneration has been recently studied using stem cells [13]. Specifically, mesenchymal stem cells (MSCs) derived from adipose tissue (AT) have been shown to differentiate into cartilage *in vitro* [14]. Additionally, liposuction, which is the surgical method to aspirate AT, has already been used in the cosmetic field and globally accepted for obtaining AT [15, 16].

The construction method of a scaffold-free three-dimensional (3D) construct by using spheroids was recently reported [8]. It is known that dissociated cells have the capacity to reaggregate through cell-to-cell attachment. These aggregates are called spheroids in this study as well as other researches [8]. Most of the approaches using these spheroids also use a specific mold to produce constructs of desired shapes [17, 18]. Certain cell types, such as fibroblasts and chondrocytes, possess the capacity to synthesize and release components of extracellular matrix (ECM); for example, collagen and proteoglycans *in vitro*. This capacity is accelerated under confluence or 3D culture conditions, because the cell cycle stops and the cells start to produce ECM components. By executing this process, we can potentially develop methods to fabricate scaffold-free 3D constructs *in vitro*.

Further, recent animal studies using rabbits and pigs presented bone and cartilage regeneration after implanting a scaffold-free 3D construct of AT-MSCs into an osteochondral defect [19, 20]. These studies were designed to evaluate autologous implantation in non-loading-bearing sites. These studies assumed that autologous implantation into osteochondral defects at these non-loading-bearing sites would allow normal cartilage to be eliminated for transplantation to occur the deteriorated sites (the loading-bearing sites). Though these studies yielded successful results, these studies should be evaluated for reproducibility, because the sample size of included animals was limited [20]. Therefore, the aim of this study was to histopathologically evaluate the regeneration of articular cartilage and subchondral bone 6 and 12 months after implanting a 3D construct of autologous AT-MSCs in a larger sample size of pigs.

## Materials and methods

### Animals

Seven cloned miniature pigs (Nippon Institute for Biological Science, Osaka, Japan), denoted as animal nos. 1–7, were used in this study. The weights (in kg) and ages (in months) of the pigs were as follows: no. (1) 28.7 kg and 28 months, no. (2) 32.5 kg and 15 months, no. (3) 29.0 kg and 14 months, no. (4) 31.1 kg and 22 months, no. (5) 33.6 kg and 18 months, no. (6) 31.6 kg and 19 months and no. (7) 38.0 kg and 25 months.

### Isolation and expansion of cells

Fifty to seventy grams of gluteal subcutaneous AT per animal was aseptically obtained by liposuction under general anesthesia. AT was suctioned using an 18-gauge Becker cannula connected with a 50-mL syringe. The collected AT was treated semi-automatically by using a tissue digestion system (The Celution^®^ system; Cytori Therapeutics Inc., San Diego, CA, USA) with proteolytic enzyme (Celase^®^TM; Cytori Therapeutics Inc., San Diego, CA, USA) [12]. Finally, 5 mL of the digested cell suspension was extracted from the system and then centrifuged at 160×*g* for 5 min at room temperature. After decanting the supernatant, the pellet was rinsed with phosphate-buffered saline (PBS) and recentrifuged. After decanting the supernatant again, the pellet was resuspended and plated in a 75-cm^2^ flask (BD Bioscience, Medford, MA, USA) in complete culture medium (CCM): Dulbecco’s Modified Eagle Medium (Gibco™^®^-DMEM; Life Technologies, Carlsbad, CA, USA) containing 10% fetal bovine serum (HyClone™FBS; Thermo Fisher Scientific, Waltham, MA, USA) and 1% antibiotic-antifungal preparation consisting of 100 U/mL penicillin G, 100 µg/mL streptomycin, and 0.25 µg/mL amphotericin B (Gibco™^®^-Antibiotic–Antimycotic; Life Technologies, Carlsbad, CA, USA). After incubation at 37 °C in 5% CO_2_ for 7 days, the cells adhering to the bottom of the dish were washed with PBS, harvested with rProtease and 1.1 mM ethylenediaminetetraacetate (TrypLE™ Select; Life Technologies, Carlsbad, CA, USA), diluted by adding 5 volumes of PBS, and centrifuged on day 7 in Passage 0. After decanting the supernatant, the pellet was rinsed with CCM, and the cells were replated at 1 × 10^6^ cells in 225-cm^2^ flasks (BD Bioscience, San Jose, CA, USA) and cultured for 6 days. The medium was changed every 3 days over 6 days in Passage 1. This serial process of passaging was repeated until the creation of a plug.

### Molecular specificity of AT-MSCs

Ten thousand cells were resuspended in 500 µL of staining buffer (SB; PBS containing 1% Fetal Bovine Serum) and incubated for 30 min at 4 °C with 20 µg/mL of fluorescein isothiocyanate (FITC)-conjugated antibodies against CD34 (Clone 581; BD Bioscience, Medford, MA, USA), CD45 (Clone 2D1; BD Bioscience, Medford, MA, USA), CD90 (Clone 5E10; BD Bioscience, Medford, MA, USA), or CD105 (Clone MEM229; Abcam, Cambridge, UK) as previously reported [20]. Non-specific FITC-conjugated mouse immunoglobulin G1κ (Clone MOPC-21; BD Bioscience, Medford, MA, USA) was used as a negative control. The FITC-labeled cells were washed with SB and resuspended in 500 µL of SB for fluorescence-activated cell sorting (FACS) analysis. Cell fluorescence was evaluated as a strong shift in the mean fluorescence intensity (MFI) on flow cytometry using a FACS Aria II instrument (BD Bioscience, Medford, MA, USA). The data were analyzed using FACS Diva software (BD Bioscience, Medford, MA, USA).

### Tri-lineage analysis

To investigate osteogenic differentiation, the AT-MSCs were placed in 6-well plates (6 Well Plate-N; Nest Biotech, Wuxi, China) in CCM at an initial density of 5000 cells/cm^2^. After 24 h of incubation, the medium was replaced with osteogenic induction medium (Differentiation Basal Medium-Osteogenic; Lonza, Walkersville, MD), supplemented with 100 µM ascorbic acid, 10 mM β-glycerophosphate, and 1 µM dexamethasone, for 2 weeks. Production of calcium apatite crystals in the osteogenic ECM was evaluated with alizarin red staining in the wells of the culture plates. In order to investigate for chondrogenic differentiation, AT-MSCs were resuspended using 6-well plates in CCM at the same density as osteogenic differentiation. After 24 h of incubation, the medium was replaced with chondrogenic induction medium (Differentiation Basal Medium-Chondrogenic, Lonza, Walkersville, MD), supplemented with 4.5 g/L d-glucose, 350 µM l-proline, 100 nM dexamethasone, and 0.02 g/L transforming growth factor beta 3, for the same period as osteogenic differentiation. The cells were stained with alcian blue to detect cartilage-specific proteoglycans. Adipogenic differentiation began when AT-MSCs reached a density of 15,000 cells/cm^2^ in the 6-well plates in CCM. Following a 24-h pre-incubation, the medium was replaced with Adipogenic Induction Medium (Lonza, Walkersville, MD), supplemented with 4.5 g/L d-glucose, 100 µM indomethacin, 10 µg/mL insulin, 0.5 mM 3-isobutyl-1-methylxanthine, and 1 µM dexamethasone, for 3 days for induction of specific molecules. Adipocyte-specific intracellular lipids were stained with oil red O.

### Preparation of three-dimensional constructs

**Fig. 1:**
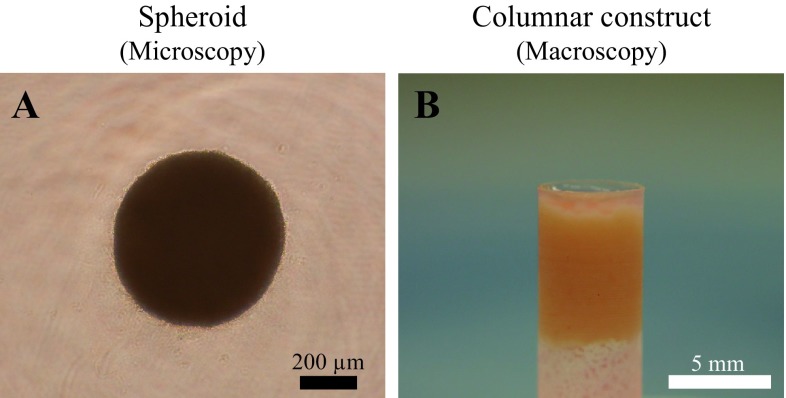
Three-dimensional construct preparation. **A** The cells formed a spheroid with a diameter of approximately 500 µm. **B** A columnar construct was created for implantation

Several methods for spheroids preparation have been introduced that, all rely on a simple common principle [8]. To allow individual cells to attach to one another, dissociated cells must be incubated in nonadhesive culture dishes, such as containers coated with nonadhesive enhanced polymers [18], alginate beads [21], and hanging drop cultures [22]. In this study, approximately 4 × 10^7^ AT-MSCs were used to produce an autologous construct. After the number of cultured cells reached 4 × 10^7^ cells, the cells were recovered and inoculated into eight 96-well plates (PrimeSurface^®^ 96 U plate; Sumitomo Bakelite Co., Tokyo, Japan) with 5 × 10^4^ cells per well. After the plates were incubated undisrupted for 48 h, the cells formed a spheroid with a diameter of approximately 500 µm at the bottom of the well (Fig. 1A). After reaggregation of the cells, each spheroid had the capacity to fuse with other spheroids. Spheroid fusion usually requires 24–72 h, depending on cell types and culture conditions, but these spheroids must be kept in culture media under controlled conditions during fusion. In this study, approximately 770 spheroids were placed into a cylindrical mold (5 mm diameter) and incubated in CCM until implantation (7 days). When the mold was carefully removed, a columnar construct with a 5 mm diameter and 5 mm height was separated and used for subsequent autologous implantation (Fig. 1B). The general outline of this construction method has been previously reported [8, 19, 20]. One of the columnar constructs was fixed in 10% neutral buffered formalin for 1 week and embedded in paraffin. Serial sections (5-µm thick) were placed on glass slides and stained with hematoxylin and eosin (HE), Safranin O, Elastica Masson, and by immunohistochemistry using specific mouse antibodies against human collagen type I (Anti Collagen I; Southern Biotechnology Associates, Birmingham, AL, USA) and type II (Anti Collagen II; Southern Biotechnology Associates) and an avidin–biotin complex system (VECTASTAIN^®^ ABC Standard Kit; Vector Laboratories, Southfield, MI, USA).

### Implantation of the constructs

**Fig. 2:**
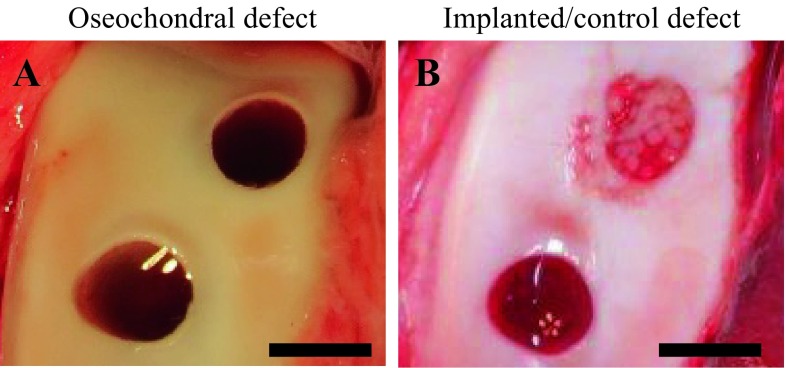
Surgical procedure. **A** Two cylindrical osteochondral defects were created in the groove. **B** A construct was autografted into the osteochondral defect in one of the two defects

The implant surgery was performed under general anesthesia using oxygen and isoflurane inhalation after pre-medication with sedatives and analgesics. One stifle was incised from the outside, and the patello-femoral groove was exteriorized. Using a bone chisel with an outer diameter of 5.2 mm, two cylindrical osteochondral defects (5 mm in depth) were created in the groove (Fig. 2A). A columnar construct (5 mm in diameter and 5 mm in height) composed of spheroids of AT-MSCs was carefully autografted into the osteochondral defect in one of the two defects (implanted defect), and nothing was implanted into the second defect (control) (Fig. 2B).

### Pathological assessment of the osteochondral defects

The pigs were sacrificed and pathologically evaluated 6 (three of the seven pigs) and 12 (the four remaining pigs) months after the surgery. Macroscopic findings of the defects in each pig were scored using the International Cartilage Repair Society (ICRS) gross grading scale [20]. The distal femurs were fixed in 10% neutral buffered formalin for 2 weeks and decalcified with formic acid for 2 weeks. Subsequently, the samples were longitudinally cut parallel to the groove and embedded in paraffin. Serial sections (5-µm thick) were placed on glass slides and stained with HE and Safranin O, and by immunohistochemistry using specific mouse antibodies against human collagen type II (Anti-hCL II; Daiichi Fine Chemicals, Takaoka, Japan) and VECTASTAIN^®^ ABC Standard Kit (Vector Laboratories, Southfield, MI, USA). The histopathological findings were also scored using O’Driscoll histological scoring (Table 1).

**Table 1:** O’Driscoll histological grading scale

Histological findings	Score
*Nature of predominant tissue*	
Cellular morphology	
Hyaline articular cartilage	4
Incompletely differentiated mesenchyme	2
Fibrous tissue or bone	0
Safranin-O staining of the matrix	
Normal or nearly normal	3
Moderate	2
Slight	1
None	0
*Structural characteristics*	
Surface regularity	
Smooth and intact	3
Superficial horizontal lamination	2
Fissures 25–100% of the thickness	1
Severe disruption, including fibrillation	0
Structural integrity	
Normal	2
Slight disruption, including cysts	1
Severe disintegration	0
Thickness	
100% or normal adjacent cartilage	2
50–100% of normal cartilage	1
0–50% of normal cartilage	0
Bonding to the adjacent cartilage	
Bonded at the both ends of graft	2
Bonded at one end, or partially at both ends	1
Not bonded	0
*Freedom from cellular changes of degeneration*	
Hypocellularity	
Normal cellularity	3
Slight hypocellularity	2
Moderate hypocellularity	1
Severe hypocellularuty	0
Chondrocyte clustering	
No clusters	2
< 25% of the cells	1
25–100% of the cells	0
*Freedom from degenerative changes in adjacent cartilage*	
Normal cellularity, no clusters, normal staining	3
Normal cellularity, mild clusters, moderate	2
Mild or moderate hypocellularity, slight staining	1
Severe hypocellularity, poor or no staining	0
Total score	24

### Statistical analysis

All numeric data were presented as the mean ± standard deviation. The differences in ICRS gross grading score and O’Driscoll histopathological scores were analyzed between the implanted defect and the control groups using Student’s *t* test. *P* < 0.05 was considered statistically significant.

## Results

### Molecular characteristics and tri-lineage potential of AT-MSCs

**Fig. 3:**
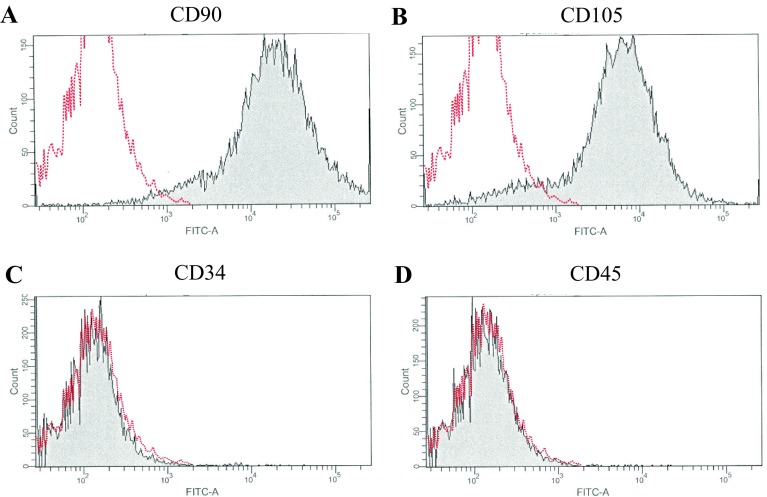
Flow cytometry results of immunological markers in AT-MSCs. A strong shift in MFI was detected with antibodies against **A** CD90 and **B** CD105. Conversely, no signal reaction was detected with antibodies against **C** CD34 and **D** CD45

**Fig. 4:**
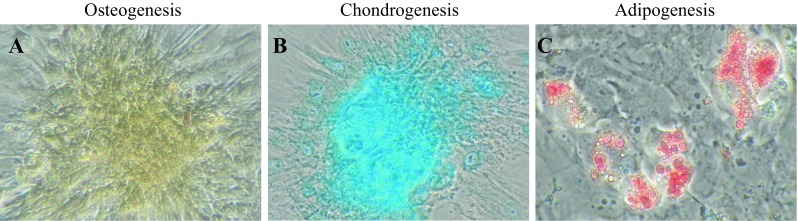
Representative images of special staining of tri-linage differentiation in AT-MSCs. **A** Following 2 weeks of osteogenic induction, MSCs showed characteristics of the ECM, including staining with alizarin red, indicating the presence of calcium apatite crystals. **B** Observation of the cells and matrix that were induced by chondrogenic induction medium for 2 weeks showed a hyaline cartilage-like structure that was positively stained with alcian blue. **C** Adipogenic induction of the MSCs resulted in adipocyte-like flattened cells with small lipid vesicles that were positively stained with oil red O

A strong shift in MFI on flow cytometry was detected with antibodies against CD90 and CD105 (Fig. 3A and B), while no signals were detected with antibodies against CD34 and CD45 (Fig. 3C and D). Following osteogenic induction, AT-MSCs aggregated and contracted to form colonies (Fig. 4A). These cells also showed appropriate characteristics of the ECM, including staining with alizarin red, indicating the presence of calcium apatite crystals (Fig. 4A). Following chondrogenic induction, AT-MSCs contracted to form colonies and showed a hyaline cartilage-like structure that was positively stained with alcian blue (Fig. 4B). Adipogenic induction of the AT-MSCs resulted in adipocyte-like flattened cells with small lipid vesicles that were positively stained with oil red O (Fig. 4C).

### Microscopic observation of the columnar construct

**Fig. 5:**
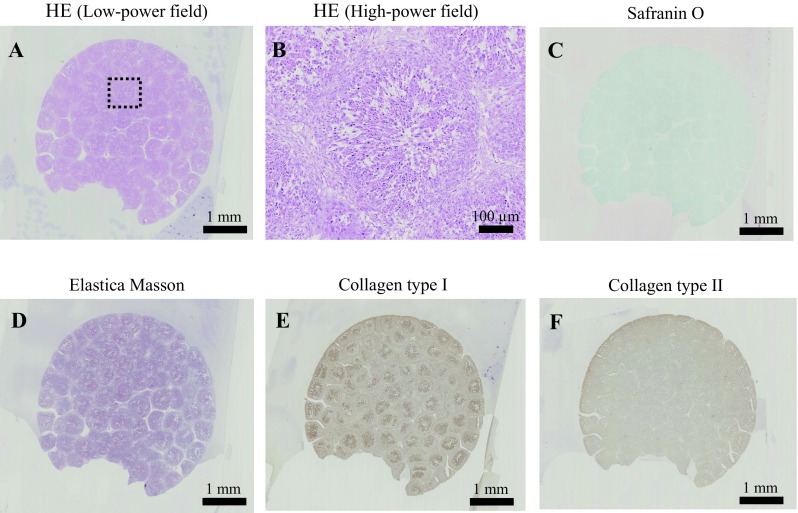
Histology of the three-dimensional construct. **A** HE staining section (low-power field) of the columnar construct. **B** Enlarged microscopic observation (high-power field) of the dotted square Safranin O staining section. **D** Elastica Masson staining section. **E** The section was stained by immunohistochemistry of type I collagen. **F** The section was stained by immunohistochemistry of type II collagen

Microscopic observation of the columnar construct is presented in Fig. 5. The boundaries of the spheroids were unclear and tended to agglutinate with each other (Fig. 5A). Numerous cell nuclei were confirmed inside and around the spheroids, and ECM production was observed but not specifically within the construct (Fig. 5B). The section stained by Safranin O was negative (Fig. 5C), but the expression of type I collagen was positively confirmed from the sections of Elastica Masson and immunohistochemistry of type I collagen (Figs. 5D and E). However, the section was not positively stained by immunohistochemistry of type II collagen (Fig. 5F).

### Macroscopic evaluation of the defects

**Fig. 6:**
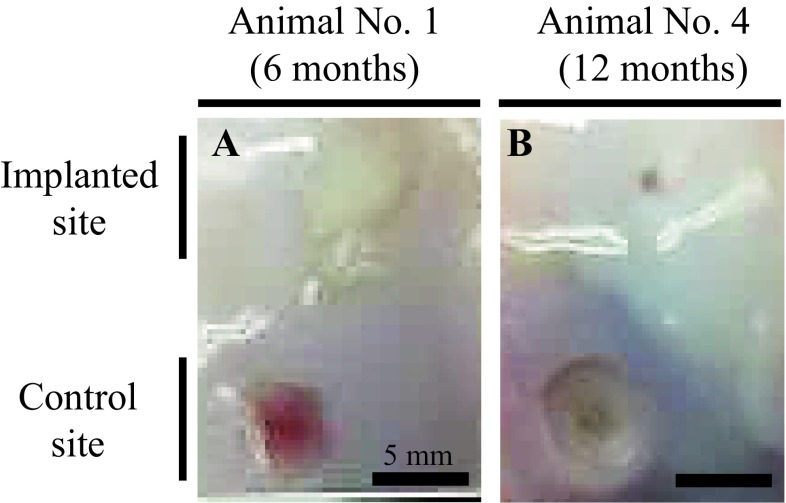
Macroscopic observations. **A** Animals no. 1 and **B** no. 4 were identified as having representative pathologies at 6 and 12 months after the implantation surgery

**Fig. 7:**
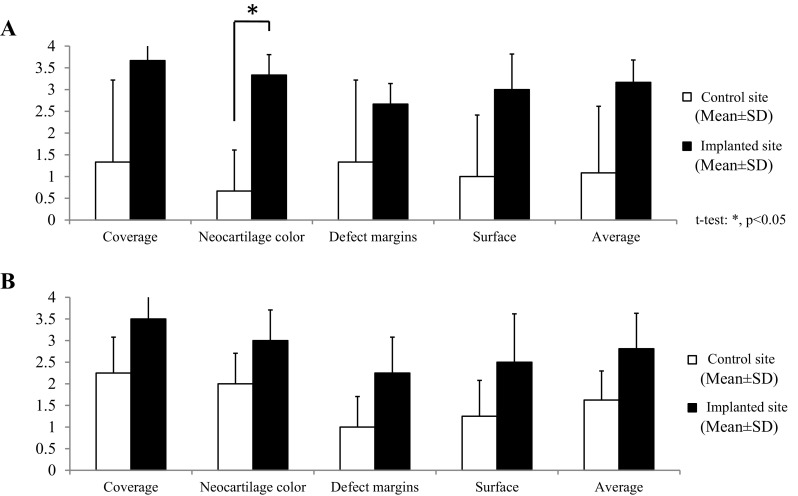
Macroscopic scores. The means of each item on the International Cartilage Repair Society (ICRS) gross grading scale at **A** 6 (three pigs) and **B** 12 (four pigs) months after the implantation surgery

**Fig. 8:**
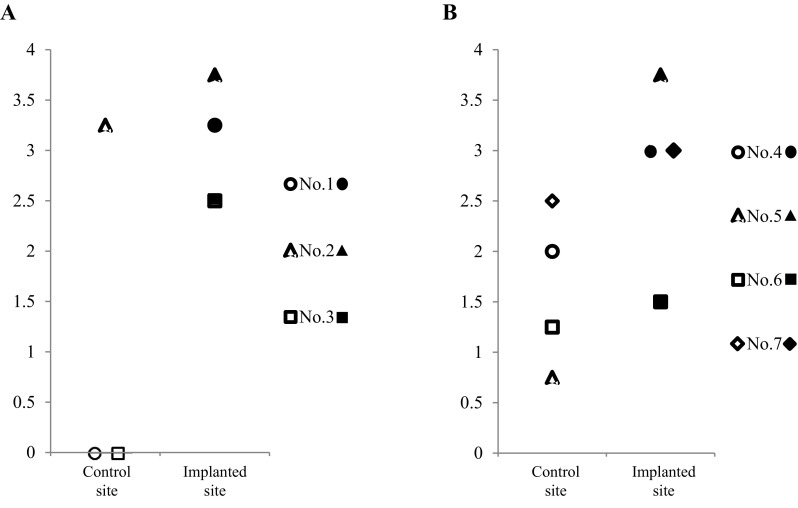
The means of total macroscopic scores for each pig by defect site at **A** 6 (three pigs) and **B** 12 (four pigs) months after the implantation surgery

Macroscopic observations of animal nos. 1 and 4 were identified as having representative pathologies at 6 and 12 months after the implantation surgery, respectively (Fig. 6). In animal no. 1 (6 months after the surgery), the surface of the implanted defect was abundantly covered with cartilaginous white tissue, while the surface of the control defect was covered with scarce cartilaginous tissue and was depressed (Fig. 6A). Conversely, in animal no. 4 (12 months of post-surgery, the surface of implanted defect was more uniformly covered with abundant cartilaginous tissue and the boundary to the surrounding normal cartilage was more unclear in the implanted defect site compared to the control site (Fig. 6B). Regarding ICRS gross grading score, the implanted defects had a significantly higher score for neocartilage color at 6 mo after the surgery compared to the controls (*P* < 0.05) (Fig. 7A); all other items were similar between the implanted defects and the controls at either post-surgery time point (Fig. 7). Furthermore, all mean macroscopic scores from the implanted defect sites were higher than the control sites at both 6 and 12 months after surgery (Fig. 8A and B).

### Microscopic evaluation of the defects

**Fig. 9:**
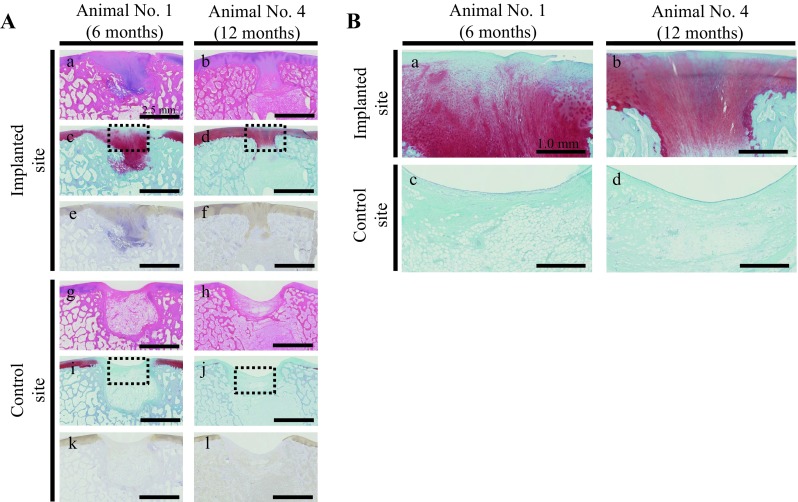
Microscopic observations. **A** Animal no. 1 was identified as having a representative histopathology (stained by **a**, **g** HE, **c**, **i** safranin-O, and **e**, **k** collagen type II immunohistochemistry) at 6 months after implantation. Similarly, animal no. 4 was representative of histology (stained by **b**, **h** HE, **d**, **j** Sarfranin-O, and **f**, **l** collagen type II immunohistochemistry) at 12 months after implantation. **B** Safranin-O staining sections were enlarged from dotted squares in (**a**) Ac, (**b**) Ad, (c) Ai and (**d**) Aj

**Fig. 10:**
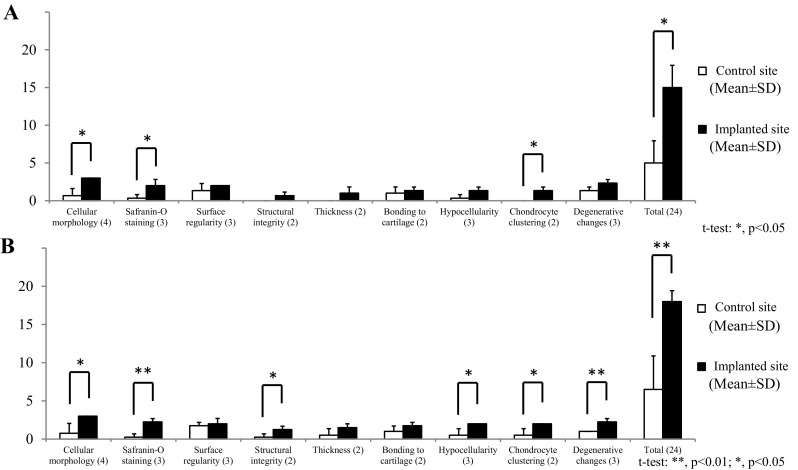
Microscopic scores. The means of each item on the O’Driscoll histological grading scale at either **A** 6 (three pigs) and **B** 12 (four pigs) months after the surgery

**Fig. 11:**
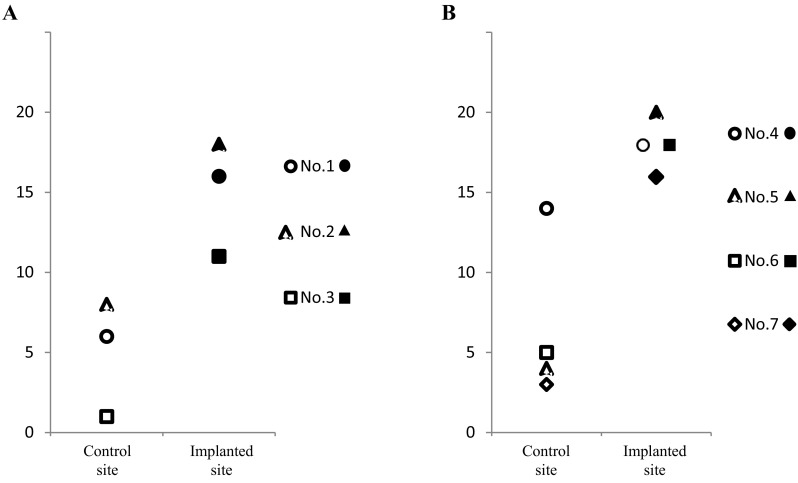
The means of total microscopic scores for each pig by defect site at **A** 6 (three pigs) and **B** 12 (four pigs) months after the surgery

Microscopic observations of animal nos. 1 and 4 were identified as having representative pathologies at 6 and 12 months after implantation, respectively (Fig. 9). Regarding the histopathology of no. 1, thick cartilage developed over the subchondral bone, the surface of the cartilage was smooth, and the boundary with surrounding normal cartilage was obscured in the implanted defect site (Fig. 9Aa, Ac and Ae). In contrast, no cartilage and subchondral bone developed, the surface was recessed, and the boundary remained in the control site (Fig. 9Ag, Ai and Ak). In no. 4, partially thickened cartilage developed over the subchondral bone, the surface was smooth, and an obscured boundary line with the surrounding normal cartilage was present, although small amounts of AT remained at the bottom of the implanted defect site (Fig. 9Ab, Ad and Af). However, in the control site of no. 4, no cartilage and subchondral bone were present, the surface was recessed, and the boundary was visible (Fig. 9Ah, Aj and Al). Moreover, the cartilage was stained more intensely and uniformly with Safranin O and Col-II immunostaining in the implanted defect site (Fig. 9Ac–Af) compared to the control site (Fig. 9Ai–Al). Furthermore, the high-power field of animal no. 1 showed that the cartilage developed to fibrocartilage in which chondrocytes and fibers were arranged irregularly at the implanted defect site (Fig. 9Ba), although ATs were packed in the defect at the control site (Fig. 9Bc). Additionally, the high-power fields of animal no. 4 showed that the cartilage matured into hyaline cartilage, in which chondrocytes were arranged cylindrically and the tide mark appeared at the implanted defect site (Fig. 9Bb), although AT infiltrated into the bony tissues at the control site (Fig. 9Bb). The total point means of the O’Driscoll histological scores were significantly higher in the implanted defects than the controls at 6 (*P* < 0.05) and 12 months (*P* < 0.01) (Fig. 10A and B). Moreover, the individual total points from the histologic scores were higher at the implanted defect sites than the controls at 6 and 12 months (Fig. 11A and B).

## Discussion

This study histopathologically evaluated the regeneration of articular cartilage and subchondral bone 6 and 12 months after implantation of a 3D construct of autologous AT-MSCs in seven pigs. In summary, this study determined that the implantation of a scaffold-free 3D construct of autologous MSCs into an osteochondral defect can induce regeneration of hyaline cartilage and subchondral bone structures over a period of 12 months.

### Molecular evaluation of the AT-MSCs and microscopic observation of the columnar construct

The swine AT-derived cells adhering to the bottom of the culture dish in this study were strongly positive for CD90 and CD105; on the other hand, CD34 and CD45 were both negative in the cells as previously reported [20]. Moreover, the osteogenic, chondrogenic, and adipogenic potential of the cells was confirmed, and we therefore defined them as swine AT-MSCs.

With regard to the microscopic observations of the columnar construct, HE staining revealed that the spheroids agglutinated with each other within the construct (Fig. 5A) and the spheroids could also be softly gripped with surgery tweezers. Moreover, numerous cell nuclei were confirmed inside and around the spheroids (Fig. 5B); however, the construct needed to be gripped carefully because ECM was present though in a small amount (Fig. 5B). The construct section was not stained positively by Safranin O due to less expression of proteoglycan, such as hyaluronic acid (Fig. 5C). On the other hand, the expression of only type I collagen inside the construct was confirmed by sections of the positive Elastica Masson staining and immunohistochemistry of type I collagen (Fig. 5D–F). Based on these results, it was confirmed that AT-MSCs secreted type I collagen in order to form a scaffold themselves, which migrated to fill in the interval spaces between each spheroid, and then formed a columnar construct before implantation. Thereafter, the cells were induced into chondrocytes *in vivo* and later secreted type II collagen (Fig. 9A). Similarly, it is possible that the proteoglycan was produced by the chondrocytes derived from AT-MSCs during the cartilage regeneration process after implantation (Fig. 9A and B). Thus, the constructs presented in Fig. 5 were thought to acquire *in vivo* characteristics and mechanical strength of the cartilage with ECM that was produced during the maturation process into osteocartilage after adhesion to the recipient tissue. Although data regarding the viability of cells and mechanical strength of constructs were not obtained in this study, they are considered to be very important for evaluation of construct properties. Therefore, these data, including the method to measure cell viability and mechanical strength of constructs, should be considered in the next study.

Moreover, we expect that this construct will be applied for the regeneration of other organs in future research. The constructs used in this study consisted of aggregates made of MSCs. The results of this study in which the implantation of the construct was implanted into an osteochondral defect showed that the deep layer of the construct was differentiated into bone and the surface layer was differentiated into cartilage, depending on the surrounding environment at the implanted defect site. It is expected that this method of implanting a construct into columnar defect can be applied to the regeneration of skin and/or meniscus. Further, by using constructs consisting of MSCs in addition to other cells, such as vascular endothelial cells and primary cells (derived from induced pluripotent stem and embryonic stem cells) [23], the construct can be applied to ocular organs (such as cornea and retina) [24–26], chest organs (such as heart and lung) [27, 28], and intraperitoneal organs (such as liver, pancreas and kidney) [23, 29, 30].

### Macroscopic and microscopic observation and evaluation of the defect sites

In this study, the macroscopic observation of the defect sites were performed (Fig. 6). In addition, macroscopic evaluations of the defect sites were completed using the ICRS gross grading score, which consisted of coverage (degree of defect filling), neocartilage color, defect margins (degree of visible circumference), and surface (degree of smooth/irregular appearance) (Fig. 7) [20].

Although the surface of the implanted defect appeared abundantly filled and smoothly covered with cartilaginous tissue at the implanted defect site compared to the control site (Fig. 6A), there was a significant difference for neocartilage color only between two defect sites at 6 months after surgery (Fig. 7A). At 12 months after the surgery, the surface of the defect was covered more uniformly and smoothly with abundant cartilaginous tissue, and the boundary to the surrounding normal cartilage was more unclear at the implanted defect site compared to the control site (Fig. 6B). Nevertheless, there was no significant difference for any mean macroscopic scores between the implanted defect and control sites at 12 months after the surgery (Fig. 7B).

When looking specifically at the ICRS gross grading scores, the means of all evaluation items at the implanted defect sites were higher than controls at 6 and 12 months after surgery. Although no significant differences were found between 6 and 12 months after surgery at the implanted defect sites, the means of some items in the controls were higher at 6 months compared to 12 months after surgery. Therefore, it is likely that the differences between the implanted defect sites and control sites tended to decrease relatively. However, all mean macroscopic scores for each animal (regardless of post-surgery time point) were higher at the implanted sites than the controls (Fig. 8A and B).

Microscopic evaluations were performed using O’Driscoll histological scores. According to the histologic scores (Fig. 10), cellular morphology, Safranin-O staining, and chondrocyte clustering were significantly higher in the implanted defects compared to the controls at both 6 and 12 months after surgery (Fig. 10A and B). These results suggest that the nature of the predominant tissue was mainly fibrocartilage at the implanted defect sites, in contrast to fibrous granulation tissue in the controls. The scores of structural integrity and bonding to the adjacent cartilage were also significantly higher in the implanted defects than in the controls at 12 months after the surgery (Fig. 10B). It is possible that both ends of the new cartilage practically bonded to the adjacent cartilage in the implanted defects between 6 and 12 months after the surgery. Conversely, in the controls, fibrous tissue, including a small amount of fibrocartilage, may have partially connected to the adjacent cartilage. The scores of hypocellularity and degenerative changes in the adjacent cartilage were also significantly higher in the implanted defects than the controls at 12 months after the surgery (Fig. 10B). Based on these results, it is suggested that degenerative changes may be milder at the implanted defect sites and the adjacent cartilage compared to the control sites between 6 and 12 months after the surgery. The total scores were significantly higher at the implanted defect sites than the controls at both 6 and 12 months after the surgery (Fig. 10A and B).

Further, the means of all items from the microscopic evaluations were higher at the implanted defect sites compared to the control sites at both 6 and 12 months after surgery. The means of multiple individual items, as well as the mean total score, at the implanted defect and control sites were slightly higher at 12 months than at 6 months after surgery. Moreover, all mean microscopic scores for each individual animal (regardless of post-surgery time point) were higher at the implanted defect sites than the control sites (Fig. 11A and B).

### Study limitations and next steps

This study confirmed that the cultured cells could differentiate into osteoblasts and chondrocytes *in vitro* (Fig. 4). However, it has not been demonstrated how to differentiate the cells in the construct into bone and cartilage *in vivo*. In order to confirm this process, the cells should be labeled and then monitored sequentially by computed tomography (CT) and magnetic resonance imaging (MRI). Unfortunately, it is technically impossible to monitor the labeled cells for a long period (longer than 6 months). Therefore, we speculate that implanted AT-MSCs may differentiate appropriately according to the surrounding environment of the implanted site. Specifically, the cells in the deep layer of the implanted construct differentiate into bone, and the cells in the surface layer of the construct differentiate into cartilage. We assume that many kinds of intercellular communication factors are involved in the process of that cell-differentiation; however, the assumption has not yet been elucidated, and the development of future research is pending.

Additionally, in this study, we could not follow-up the process of osteochondral regeneration via imaging techniques such as CT and MRI. Continuous imaging using CT and MRI is essential to observe the process and the degree of osteochondral reconstruction *in vivo*, and the data obtained using these imaging techniques enable calculation of bone and cartilage volume. Therefore, CT and MRI data should be obtained, and the osteochondral regeneration process should be evaluated objectively in a future study. However, the results in this study suggest that there is an increased progression of cartilage regeneration between 6 and 12 months after implantation.

We hypothesized that artificial scaffold may be unfavorable to regenerate articular cartilage. In previous research, many kinds of artificial scaffolds, such as hydrogel with and without chondrocytes and/or stem cells, have been implanted into cartilage defects [31–33]. Unfortunately, it has not yet been reported that any kinds of artificial scaffolds with cells have regenerated smooth hyaline cartilage at the cartilage lesion in large animals. It is unclear whether reliable methods to regenerate articular cartilage can mimic the ways in which cells, biomaterials, or tissue engineering are used for a long time [34, 35]. Therefore, we plan to compare artificial scaffolds including cells with only MSCs to confirm if artificial scaffold is suitable for regeneration of hyaline cartilage.

Finally, of most importance, it is necessary to determine whether the same method can be extrapolated to other animals, including humans. Our research group has already attempted and confirmed that it is possible to produce the same constructs as this study using human stem cells (data not shown). We are currently preparing another paper to report further details on human experiments. We are convinced that it is possible to replicate osteochondral regeneration by implanting the stem cell construct in other animals and even into human patients.

This study concluded that implantation of a scaffold-free 3D construct of only AT-MSCs into an osteochondral defect was able to regenerate the original structure of the cartilage and subchondral bone in a large animal.
